# Tympanometry as a predictor factor in the evolution of otitis media with effusion

**Published:** 2012-12-25

**Authors:** C Oprescu, M Beuran, AE Nicolau

**Affiliations:** * “Medical Center for Diagnosis and Treatment”, Bucharest, Romania; ** “Carol Davila” University of Medicine and Pharmacy, Bucharest, Romania, “Institute of Phonoaudiology and Functional ENT Surgery”, Bucharest, Romania

**Keywords:** otitis media with effusion, tympanometry

## Abstract

Background: Otitis media with effusion (OME) is one of the most frequently met pathologies in small children. Long-term persistence of the liquid in the middle ear cavity correlates with the impairment in speech acquisition and poor results in school.

Aim: To evaluate the predictive value of impedancemetry in recovery of the normal middle ear status.

Methods: 30 children (age 4 month - 9 years) with OME were periodically monitored by means of tympanometry. The children were treated with the same treatment protocol for 7 days minimum and tympanometry was repeated after seven and fourteen days. After follow-up tympanometry at 7 days, children with abnormal middle ear condition were randomly allocated into two groups: one, which continued the same treatment for another 7 days and one group with no treatment for the next 7 days.

Results: After 7 days, 64% of the patients had an improvement in tympanometry (type C tympanogram) and 10% had a complete resolution of the middle ear effusion (type A tympanogram). After 14 days, tympanometry was normal in 74% of the patients (53.9% rate of success in the no-treatment group).

Conclusion: Complete resolution of the middle ear effusion is obtained in various periods of time, depending on numerous factors, with an appropriate treatment. Tympanometry proved to be a good tool in predicting the length of the treatment.

## Background

Otitis media with effusion (OME) is defined as an inflammation of the middle ear mucosa along with an accumulation of liquid without signs or symptoms of acute infection (especially fever). The criteria for diagnosing OME refer to the presence of a fluid in the middle ear without signs of acute infection and without another underlying medical condition. If the liquid persists for more than three weeks, we consider it a chronic otitis media with effusion as diagnostic.

Clinical diagnosis is performed through otoscopy with the visualization of the fluid, which may present characteristics of plasma exudation or of mucus secreted by mucus secreting cells. In the first case, the tympanic membrane remains translucent, and the presence of blisters or the level of liquid may be verified, in addition to the degree of retraction. In the second case, there is loss of translucency of the eardrum, with a frequent increase of its radial vascularization. 

Tympanometry is an excellent diagnostic test, with 85% specificity in cases of middle ear secretion, in which it shows an increased impedance in the propagation of sound by the tympanic-ossicular chain complex, measured and recorded in a tympanogram [**1,13-15**].

One of the earliest uses of tympanometry was to estimate the middle ear pressure and, indirectly, to measure the Eustachian tube functions because normal Eustachian function is necessary for the maintenance of normal middle ear pressure. Middle ear pressure is now routinely estimated from the tympanogram. TPP is the pressure at which the peak of the tympanogram occurs and is assumed to be the point at which the pressure in the ear canal equals the middle ear pressure. Therefore, TPP is an estimate of the pressure that provides the greatest admittance, or least impedance, to the flow of acoustic energy into the middle ear. Related to the measure of middle ear pressure is the measurement of the Eustachian tube function. The major reason to assess the Eustachian tube function in patients with intact tympanic membranes is the association of the Eustachian tube dysfunction and otitis media. Deviations in TPP from the atmospheric pressure can suggest a disorder of the Eustachian tube, which can be associated with middle ear effusion. In a study of children scheduled for myringotomy, negative pressure peaks on tympanograms were related to a high incidence of the recurrent acute otitis media with effusion. Further, the more negative the pressure peak was, the more likely the child was to suffer from repeated episodes of middle ear effusion cautions, because there are different diagnostic implications for a flat tympanogram with a severe negative peak: a wide pressure range should be used to verify that there is not a peak at a pressure beyond the pressure range, that is normally evaluated [**1,14,16**].

TPP has also been used to monitor the development and resolution of otitis media. In the earliest stages of otitis media, the TPP may be positive and become negative as the infection progresses. One theory for the negative pressure is that the air in the middle ear is absorbed by the surroundings tissues, resulting in negative pressure. Long-term negative pressure may be followed by an accumulation of fluids in the middle ear, resulting in an effusion and a flat tympanogram. Therefore, abnormal pressures can provide an early warning of a developing otitis media. Theoretically, early identification of the middle ear disease can lead to an early medical referral and treatment of the disease, before the condition becomes chronic. Subsequently, as the disease resolves, the tympanogram changes from flat, to negative peak, and then to normal peaked [**1,2,13-16**].

The audiometric testing can reveal decreased hearing, with air-bone gaps in the low frequencies, 250 to 1000Hz. The inset audiogram shows the wide range of hearing losses measured in the patients when the tympanograms were flat. 

Tympanometry is usually performed with 226Hz probe, but high-frequency tympanometry (1000Hz) is mandatory in infants below 6 months in order to avoid false-positive results.

More than that, studies showed that High-frequency tympanogram is advantageous in identifying mass-related pathology. Measures of the tympanogram width (TW) and peak compensated static admittance at 226 Hz can also indicate an abnormality, but the use of a high–frequency probe tone typically accentuates the abnormality by showing a broad, shallow notch that is obvious from a simple visual analysis of the tympanogram [**2,3,11-15**]. 

Middle ear effusion is associated with conductive hearing loss. Its persistence in both ears is responsible for the long-term consequences like impairment of speech development and other cognitive disabilities [**6-10,15,17**].

## Methods

30 consecutively children, aged 4 months to 9 years were included in the study, 17 boys and 13 girls. OME was diagnosed based on the medical history, otoscopic examination, and tympanometry.

 Parents filled in a questionnaire on the child’s history of ear infections, atopic or allergic diseases, snoring, adenoidectomy and tympanostomy tube insertion, day-care attendance, baby’s usage of the pacifier and their own smoking habits.

 The children were treated with the same treatment protocol for minimum 7 days and tympanometry was repeated after seven and fourteen days in all of them. After follow-up tympanometry at 7 days, children with abnormal middle ear condition were randomly allocated into two groups: one, which continued the same treatment for another 7 days and one group with no treatment for the next 7 days. Tympanometry was repeated after other 7 days.

 Resolution of OME, the primary endpoint in the trial, was defined as a conversion from a B-type curve to an A-type curve in tympanometry, monitoring for at least two consecutive days. 

 The duration of OME in each group was analysed as a primary outcome.

## Results

The initial clinical diagnosis was bilateral OEM in 20 children (66.67%), right sided in 7 (23.33%) and left sided in 3 (10%).

 After 7 days, 50% of the patients had an improvement in tympanometry (type C tympanogram) and 10% had a complete resolution of the middle ear effusion (type A tympanogram). The 27 children with persistent abnormal middle ear function after 7 days of treatment were randomly allocated into two groups:

 • 14 children continued the same treatment for another 7 days

 • 13 children discontinued the treatment and they were evaluated again after 7 days

 After 14 days, tympanometry was normal in 93% of the patients who were still with abnormal tympanometry result at 7 days check-up, mostly (51.85%) from the group with 14 days treatment. Success rate in the group with no treatment in the last 7 days was just 46.1% and 100% in the group with 14 days-treatment.

 From 10 children with unilateral OME, 3 had normal tympanometry after 7 days of treatment and the other 7 had normal tympanometry after 14 days, regardless the group they were allocated to. We can say that children with unilateral OME cured more rapidly than those with OME in both ears. The 8 children with OME and type B tympanometry after first 7 days of treatment were younger (less than 1 year of age) than those recovering faster.

 There were 4 children (13.33%) with primary unilateral OME, in whom liquid appeared in the opposite healthy ear during the treatment, but they were normal in both ears at 14 days check-up.

 Symptoms that correlated with the lasting of middle ear effusion were rhinitis and enlarged adenoids.

## Discussion

Monitoring the middle ear status by means of tympanometry is important in the prediction of the child’s OME evolution. Depending on the result after the initial treatment, we can recommend further treatment or not, based on objective results – the tympanogram. 

 More frequently, the monitoring by tympanometry (even in periods with no clinical suspicion of hearing loss) also enables the knowledge of the response of the individual patient to treatment, in order to further decide the surgical treatment (adenoidectomy or transtympanal ventilation tubes).

 As children younger than 2 years old have a higher risk of bilateral OME from the point of view of symptomatic and audiological failure than older children, age is a confounding factor [**4,6,7**]. A periodical monitoring by impedancemetry was successful and gives us information about the recovery process and the resolution of effusion [**15**].

 Being a well-documented method for the diagnose of OME, tympanometry has a sensitivity varying between 82–90% and a specificity between 68–98% relative to findings in myringotomy [**4,5,12**].

 A peak tympanogram, type A or C eliminates the hearing impairment due to OME with 98% certainty and thus the method can be used to assess the main risk of the long-term complications [**2,3,13,15,16**].

 Our paper reveals that the periodical monitoring of the middle ear status by means of tympanometry is a very useful objective tool in predicting the evolution of the OME and the length of the treatment, since we observed a 100% rate of success in resolving the OME in patients who continued the treatment as if they had an abnormal middle ear function after 7 days of treatment and just 46.1% success rate if they stopped the treatment before achieving a normal middle ear function, similar with the patients with not-long enough treatment, since tympanometry is not performed during the treatment.

## Conclusions

Tympanometry has proved to be a valid method for diagnosis of the OME and we consider it has a good clinical value as a predictor factor for the length of the appropriate treatment of the OME, in order to obtain a normal middle ear function.
